# Neuronal Adaptation Translates Stimulus Gaps into a Population Code

**DOI:** 10.1371/journal.pone.0095705

**Published:** 2014-04-23

**Authors:** Chun-Wei Yuan, Leila Khouri, Benedikt Grothe, Christian Leibold

**Affiliations:** 1 Department Biologie II, Ludwig-Maximilians-Universität München, Planegg-Martinsried, Germany; 2 Department of Neurobiology, The Hebrew University of Jerusalem, Jerusalem, Israel; The University of Plymouth, United Kingdom

## Abstract

Neurons in sensory pathways exhibit a vast multitude of adaptation behaviors, which are assumed to aid the encoding of temporal stimulus features and provide the basis for a population code in higher brain areas. Here we study the transition to a population code for auditory gap stimuli both in neurophysiological recordings and in a computational network model. Independent component analysis (ICA) of experimental data from the inferior colliculus of Mongolian gerbils reveals that the network encodes different gap sizes primarily with its population firing rate within 30 ms after the presentation of the gap, where longer gap size evokes higher network activity. We then developed a computational model to investigate possible mechanisms of how to generate the population code for gaps. Phenomenological (ICA) and functional (discrimination performance) analyses of our simulated networks show that the experimentally observed patterns may result from heterogeneous adaptation, where adaptation provides gap detection at the single neuron level and neuronal heterogeneity ensures discriminable population codes for the whole range of gap sizes in the input. Furthermore, our work suggests that network recurrence additionally enhances the network's ability to provide discriminable population patterns.

## Introduction

Behaviorally relevant auditory signals such as speech, or the reverberations that convey information about the spatial environment, are characterized by temporal features in the lower millisecond range. The intrinsic time scales of neurons that represent the auditory information in the downstream cortical processing centers are, however, much slower [1], [2]. The general view of the auditory pathway is thus that it translates the temporal code of the acoustic wave into the population code of the cortex, and relaxes the required temporal precision of cortical processing to the time scale of tens of milliseconds [3]–[5]. This translation between time and rate representation is assumed to gradually occur along the multiple processing centers in the auditory brainstem [6], [7].

A central stage in the ascending auditory pathway is taken by the inferior colliculus (the auditory midbrain), which collects most afferent projections and transfers them to the thalamo-cortical system [8]. In this sense the inferior colliculus acts as a hub, meaning that most auditory information processed by cortical centers has to be somehow represented in the inferior colliculus. The neurons in the inferior colliculus are characterized by a large diversity of in vivo responses [5], [9], [10] and cellular parameters, in particular temporal ones such as onset vs. sustained firing [11], membrane time constants and adaptation currents [12]. It is therefore reasonable to assume that the inferior colliculus population represents acoustic information in both spike timing and rate [13], [14]. Moreover, one expects the rich assortment of neuronal behaviors observed at the inferior colliculus to play a central role in the computational capacity of the population code.

In this paper, we investigate the transformation from a temporal to a population representation using the simple paradigm of gap stimuli. We re-analyzed in-vivo recordings from anesthetized gerbils to show that such transformation indeed takes place at the level of the inferior colliculus. We then construct a computational model suggesting that the heterogeneity of biophysical properties of the neurons, particularly of their adaptation time constants, can explain the in-vivo phenomenology.

## Materials and Methods

### Ethics Statement

All experiments were approved according to the German Tierschutzgesetz (AZ 55.2-1-54-2531-57-05 Regierung von Oberbayern). For more details see original data publication [15].

### Data Analysis

**Figure 1:**
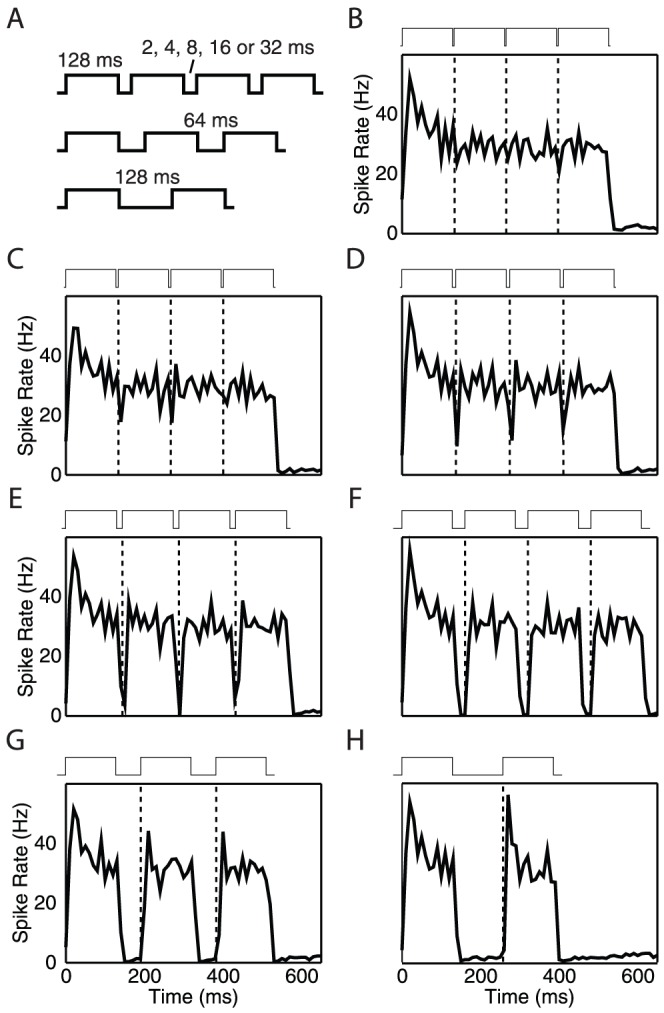
Gap stimuli and network rate response. (A) Schematics of the gap stimuli used in the experiments. Each pulse is 128 ms long and contains white noise between 0.5 and 12 kHz. For the 2, 4, 8, 16 and 32 ms gap sizes, 3 gaps are presented per stimulus. For the 64 ms gap stimulus, 2 gaps are presented, whereas only a single 128 ms gap was placed in the pulse train. (B) Mean firing rate of the gerbil inferior colliculus network (91 neurons) in response to the 2 ms-gap stimulus. Bin size  = 10 ms. The dashed lines mark the locations of the gap-to-pulse interfaces. (C)–(H) Mean network firing rate in response to the 4, 8, 16, 32, 64, 128 ms-gap stimuli.

We re-analyzed previously published single unit recordings from 91 inferior colliculus neurons of young adult Mongolian gerbils with best frequencies from 2 to 12 kHz [15]. Each stimulus was composed of a series of symmetric, broadband (500 Hz to 12 kHz) sound pulses of 128 ms duration interjected with silent intervals (gaps) of a fixed length, as shown in Figure 1A. The pulse-gap interfaces used in the experiment were ramped with 1 ms rise and fall times. These ramps are assumed to be negligible compared to the duration of the sound pulse (128 ms) for further analysis of the population code, hence these ramps are shown as steps in the schematics of Figure 1A. Between stimuli, the gap lengths range exponentially from 2 to 128 ms (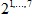 ms). Therefore, each stimulus pulse train is characterized by the particular gap length it carries.

Due to the limit on the total length of the stimulus, the number of times the sound pulses are repeated per pulse train varies, as illustrated in Figure 1A. The resultant pulse trains were presented to the anesthetized animal through ear phones, and each neuron was recorded over multiple (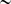 10) trials of the same pulse train stimulus. For more detailed description of the experimental procedures, we refer to [15].

The mean population rate response to each stimulus is shown in Figures 1B–H. The network response to the stimulus typically follows a transmission delay. By inspecting the ramp-up of the population rate response relative to the first pulse in the stimulus, we consistently found this latency to be 12 ms across different gap stimuli. This transmission delay is already applied in Figures 1B–H, where time  = 0 denotes the onset of the network's reaction to the first (control) pulse of the stimulus (gap size  =  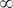), and the dashed lines represent the gap-to-pulse interfaces. All single-neuron spike times for later analyses are latency-corrected according to this transmission delay.

The spike times were translated into post-stimulus time histograms 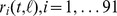 (bin size 10 ms) by averaging over all repetitions of post gap activity snippets, where 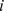 denotes the neuron index, 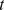 denotes the post-gap time, and 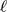 denotes the gap length.

### Independent Component Analysis

For our independent component analysis (ICA), we employ the FastICA algorithm [16] on the vectors 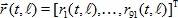 considering all combinations of 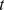 and 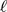 as single measurements. As a means of noise-filtering, ICA is applied on a low-dimensional subspace identified by the number 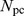 of principal components of the full data set of 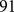 dimension. PSTHs in this low dimensional space are denoted as 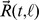, i.e., every PSTH vector 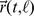 is approximated by a linear superposition of 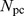 ICs 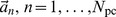,
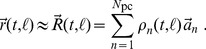
(1)


The ICs are normalized, 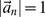, and 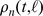 denote the projections to the subspace spanned by 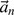. Note that 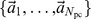 do not necessarily form an orthogonal basis set and thus the projections are calculated as 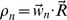, using the dual basis 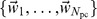 defined by
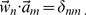



An important step is to find the minimum value of 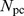 that captures all gap-sensitive components. To determine this value, we begin with 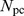  = 1 and examine the resultant independent component. We then increment 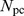 by 1 until we reach a final 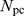 beyond which no more gap-sensitive ICs can be identified by visual inspection. For most analyses we thereby obtained 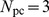, explaining 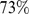 of the data variance. This approach allowed us to extract all gap-sensitive ICs that possess enough signal strength.

### Neuron Model

As a neuronal model, we use the integrate-and-fire neuron, where the membrane voltage 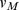 integrates exponentially-decaying synaptic currents (see Section Synapse Model). Simulations are performed using the Neural Simulation Technology (NEST) Initiative software package, version 2.0 [17], at a time resolution of 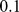 ms.

The membrane time constant (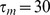 ms) and membrane capacitance (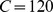 pF) are taken from the mean experimental values of [18] unless otherwise mentioned. The membrane potential thus follows the dynamics
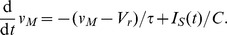
where 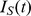 denotes the synaptic current (see below). The resting potential (and reset potential) of every neuron is 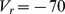 mV, while the spike threshold is set to be 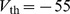 mV. These values are only used for convenience of illustrations in the Figures. The values are effectively irrelevant for the neuron model used (integrate and fire with current-based synapses), since their difference only acts as a scaling factor for synaptic strengths. After a spike, the membrane voltage 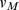 is reset to the resting potential and the neuron goes through a refractory time of 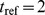 ms (limiting the maximal firing rate to below 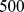 Hz), before post-synaptic currents are integrated again.

Adaptation is implemented as an exponentially-decaying hyperpolarizing potential 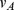 that follows the dynamics
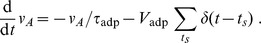



Each spike (at time 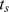) decrements 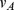 by 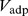  =  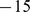 mV and 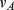 decays back to zero with a time constant 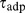 that may be different for each neuron. This adaptation effect is additive; hence, the resulting adaptation potential
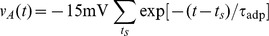
and the membrane voltage 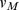 are evaluated separately and summed up afterwards to be compared to the threshold 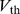.

### Network Topography

For our standard network, we use the following parameters: 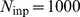 inputs are feed-forwardly directed to a network of the same size (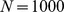 neurons). Each input fiber projects to a small random fraction of 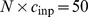 network neurons, where 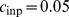 is the input connectivity. Also the recurrent network connectivity 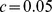 is sparse: each network neuron is connected to 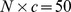 other network neurons. Thus, the total impact of feed-forward and recurrent connections is balanced.

In some simulations we use different values of 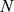, 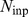, 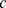, and 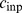 as indicated.

### Synapse Model

Synaptic currents
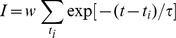
are evoked by input spikes at times 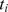, and decay exponentially with time constants 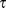 of 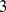 ms for inhibition and 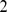 ms for excitation as measured in [18].

For the feed-forward input to the network, the excitatory synaptic weight is set to be 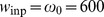 pA, roughly half of what is needed to bring a neuron to threshold from resting potential. Within the network, the weight of recurrent excitation 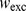 is measured in units of 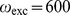 pA/ 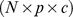, where 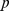 is the fraction of the excitatory neurons in the network. For inhibition, the weight 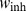 is given in units of 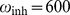 pA/ 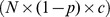


All synaptic transmissions introduce an additional delay of 1 ms, which is a typical value in many modelling studies.

### Linear Classifier

To test how well the network activity discriminates between different gap sizes in the input we trained a linear classifier. The performance of the classifier on the test set (test accuracy) is used as a criterion for discriminability. As a linear classifier we use the LIBSVM support vector machine implementation provided by the SHOGUN machine learning toolbox [19].

We began by constructing 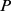 unique pairs of spike trains snippets. Each snippet pair was then used to build 2 input patterns, one with gap size 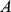 and the other with gap size 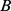. The resultant input patterns were fed to the network to train the classifier. Each output vector was generated by counting the spikes in the time bin corresponding to the onset of the 2nd snippet, where the bin size was chosen to be 30 ms to match the average time constant of the cell membrane [18]. Once the classifier was trained using the output vectors, we shuffled the order of the original input patterns and laid these shuffled patterns over a new background noise. This “test input” was then streamed into the same network for a new set of output vectors, and the accuracy at which the previously trained classifier identified the gap sizes associated with each output vector was used as the quantity to gauge the network's capacity to encode gaps. To avoid over-fitting, we keep 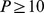 such that many realizations of each gap size are processed. For each parameter set, the experiment was repeated 100 times to gain statistical significance.

## Results

### Population Coding of Gaps in Gerbil inferior colliculus

**Figure 2:**
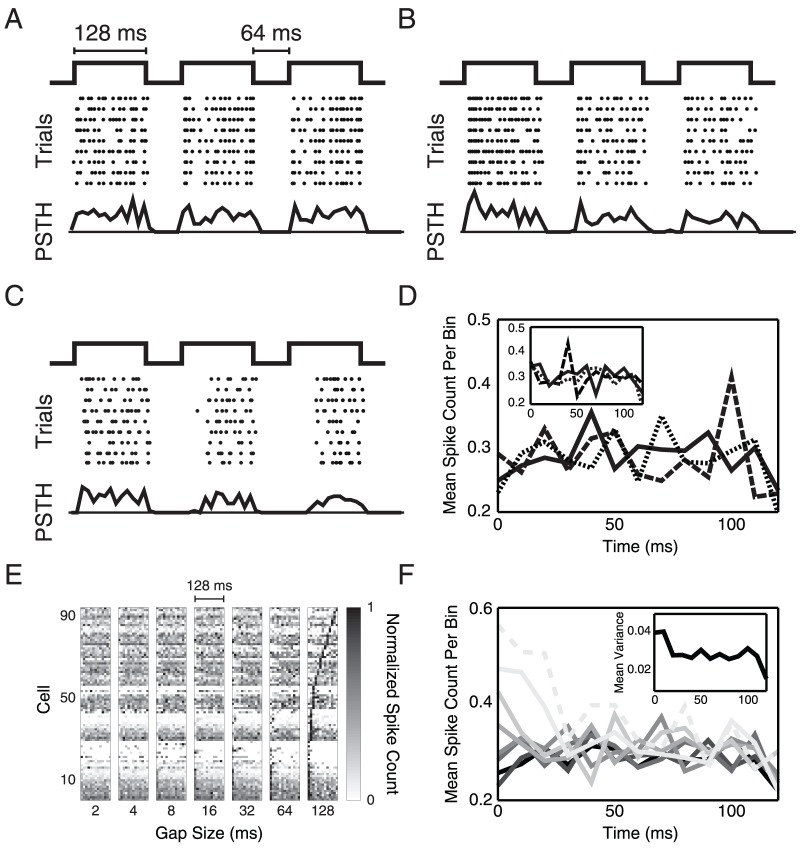
Population rate response to gap sizes. (A)–(C) Exemplary gerbil inferior colliculus neurons and their responses to repeated trials of the same stimulus. The stimulus is comprised of three 128 ms broadband pulses that are separated by two 64 ms silent intervals (gaps). The resultant trial-averaged post-stimulus time histograms (PSTHs) are generated with a 10 ms bin size. The first two neurons show fast onset responses, while (C) shows delayed onset behavior. (D) The mean network PSTH during the first (solid line), second (long dashed) and third (point dashed) post-gap pulse of the 2 ms gap stimulus. One sees no clear pattern as a function of pulse-repetition. Inset: the same mean PSTHs for the 32 ms gap stimulus. (E) Grey level plot of cell-wise normalized post-gap PSTHs for all 91 cells and all gap sizes obtained from averaging over all pulses in the train following a gap. The cells are ordered according to their PSTH peaks for the 128 ms gap stimulus. (F) Mean network spike count over the 13 bins for each gap size. Dark to bright means short to long gap sizes. The dashed line is the mean network response during the first pulse, i.e. the control response. Inset: the mean network spike count variance over all gap sizes during the post-gap time series. The dips in the last bin reflect the fact that it only contains 8 ms of stimulation for a 10 ms bin size.

Temporal features of auditory stimuli on the millisecond scale are preserved in the time course of the firing rates of inferior colliculus neurons [15]. To see whether they are represented as population patterns in the inferior colliculus as well, we performed a population rate analysis (see Materials and Methods section on ICA). The underlying data are illustrated in Figures 2A–C, which show two typical neuronal responses to a pulse train with 64 ms gaps. Each neuron was measured during multiple trials of the same stimulus, and the resultant latency-corrected post-gap spike times were binned and averaged to render a post stimulus time histogram (PSTH) representing this neuron's response to the stimulus, as illustrated at the bottom in Figures 2A–C. Because gap-encoding necessarily occurs after the presentation of the gap, only those spike counts during the 2nd and latter pulses were considered for further analysis. For each cell, the sets of post-gap PSTH were averaged to improve signal-to-noise ratio. This averaging across pulse-responses is justified because we observed no discernable pattern arising as a function of pulse-repetition in our data (Figure 2D and inset). Hence, for each gap size, we obtained a population spike count raster matrix as shown in Figure 2E.

For each gap size in Figure 2E, we obtained the network-averaged spike count and its variance as a function of time (Figure 2F). These results suggest that (1) the network encodes gap size by the neuronal spike rates immediately following the gap, with large gap sizes eliciting high rate responses and vice versa, and (2) gap size is encoded in the first 30 ms after the gap, and beyond this point, the network has reached a steady balance between external stimulation and intrinsic activity. To extract the underlying patterns from the noisy data, we collected the first 70 ms of each spike count raster matrix in Figure 2E and concatenated them for independent component analysis (ICA).

**Figure 3:**
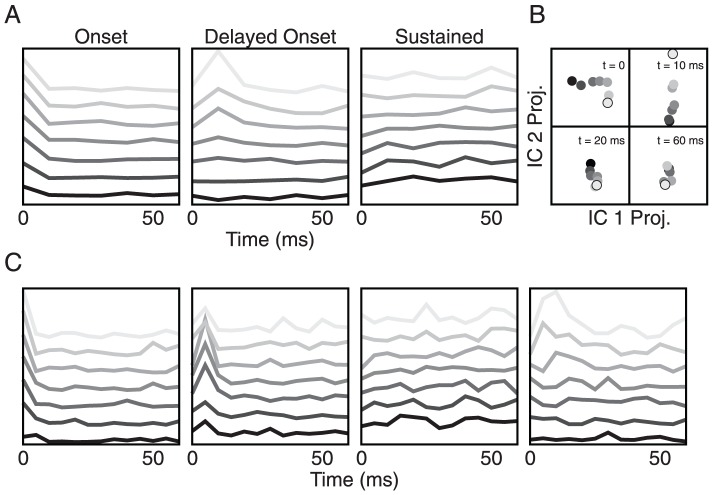
Gap-encoding network patterns. (A) Independent component analysis (ICA) of the population response matrices in Figure 2E, taking only the first 70 ms of each neuronal response. ICA reveals 3 significant independent components (ICs) that can be interpreted as onset, delayed onset and sustained (dark to bright means short to long gap sizes). All three vertical axes possess the same scale and, for illustration purposes, the baseline values for the different gaps are shifted equidistantly relative to each other. (B) Projections onto the subspace spanned by the onset and the delayed onset components for different points in time as indicated. Based on these two components it is possible to distinguish the responses to different gap sizes (gray levels as in A) for a few tens of milliseconds after the onset of the subsequent noise pulse. (C) ICs from analyzing our neuronal spike data with 5 ms bin size.

ICA found 3 population patterns (explained variance: 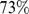) that correspond to well-known inferior colliculus response types: onset, delayed onset, and sustained (Figure 3A). Our ICA procedures (see Materials and Methods) indicated the onset response to be the most predominant component, followed by the sustained and the delayed onset component. Beyond 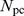  = 3, no more discriminant patterns were found. While the sustained component yields no discriminability, the onset and the delayed onset components clearly encode gap sizes early on during the post-gap network response with the same rate-encoding mechanism, and the separation is lost during the latter part of the pulse (Figure 3B). These ICA results are consistent with the firing rate responses of Figure 2F. In fact, the onset and the delayed onset components likely originate from the same population dynamics, and their differentiation stems only from binning. In Figure 3C, we applied the same analytical procedures to our data, this time with 5 ms bin size, to reach 4 independent patterns (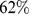 explained variance), 3 of which display gap-discrimination. Comparing the gap-encoding patterns of Figure 3A to their counterparts in Figure 3C, we observe two delayed onset components indicating a continuous temporal code of the gap sizes. One thus may generalize that gap-discrimination in these gerbil inferior colliculus neurons arises from one predominant mechanism: the time course of activity within 30 ms post-gap. Generally, larger gap sizes are encoded by higher network firing rate. Upon closer inspection of Figure 2F, a plausible neurophysiological explanation is that after a longer gap the cells' excitability has recovered better than after a shorter gap. Along these lines, the fact that these neurons fire briskly after a long gap before dropping to a lower, steady-state rate also points to the possibility of adaptation in the network.

### Simulation Paradigm

**Figure 4:**
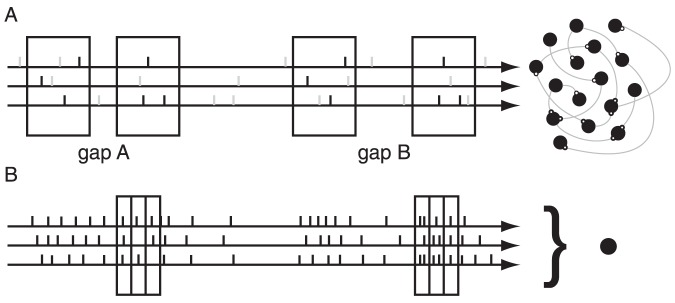
Gap discrimination paradigm. (A) Schematic of the input stream to the network. Sensory-evoked spikes (black ticks) from Poisson processes (3 shown) and spontaneous background spikes (gray) are fed into a network. Each box marks a 130 ms snippet, and the gap size is defined as the silent interval (with noise) between the two snippets. Two input patterns, with identical snippets (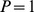) and differing only in the gap sizes (gap A and gap B), are shown in a single input stream, with a 900 ms spacing between them. (B) Schematic of the network's output is read out at the onset of the second snippet with a bin size of 30 ms (the first black box, latency-corrected). The output patterns are translated into population vectors of spike counts and then used to train a linear classifier (filled circle) to distinguish the gap A vectors from the gap B vectors. Later on, when we perform ICA on simulated networks, the bin size is switched to 10 ms to collect 13 bins from the second snippet.

To gain a mechanistic understanding of how such a population code may be generated, we devised a simple computational network model (see Materials and Methods and Figure 4A), which was inspired by our previous work [20]. The network consists of 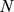 integrate-and-fire neurons with simple adaptation behavior. These neurons receive input from 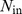 input fibers with low connectivity 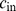. The input spikes are supposed to mimic the activity evoked by the noise pulses. Focusing on high-frequency channels of the auditory pathway (recorded IC neurons had best frequencies above 2 kHz) in which phase-locking is absent, we assume that there is no inherent temporal structure in the input spike trains and model them as Poisson processes. Each input pattern is comprised of a silent interval of a certain length, surrounded by two snippets of such neuronal population spike trains. All snippets are of identical duration (130 ms) and all fibers fire at an identical mean rate (Poisson density) of 10 Hz, unless otherwise stated. In addition, ongoing spontaneous background spikes (noise) are imposed along each nerve fiber according to a second independent Poisson process. For simplicity, patterns are presented with a 900 ms spacing in-between to avoid serial correlations.

In response to the temporal input patterns, the network produces temporally distinct rate patterns that encode the different gap sizes (Figure 4B). These rate patterns are directed downstream to a model of the thalamo-cortical system for read-out. We employ a linear classifier as a stand-in for the thalamo-cortical read-out, as it could be easily represented by neural elements and requires the fewest assumptions about the read-out structure. In other words, we use the linear separability of the output population patterns as a benchmark to evaluate the network's ability to encode different gap sizes. While it is possible that the thalamo-cortical system implements a non-linear classification algorithm, such a criterion would be much more prone to overfitting. Conversely, linear classification has the advantage of being a very conservative measure for discriminability.

Under these assumptions we optimized the network's gap-discrimination performance with respect to adaptation variables and recurrence strengths. More specifically, we studied the network using a binary classification task, where the network is asked to distinguish between two different gap sizes (see Materials and Methods, Linear Classifier).

In the following, this paradigm will be used to evaluate the influence of cellular and network parameters on discrimination performance.

### Single Neuron Gap Discrimination

**Figure 5:**
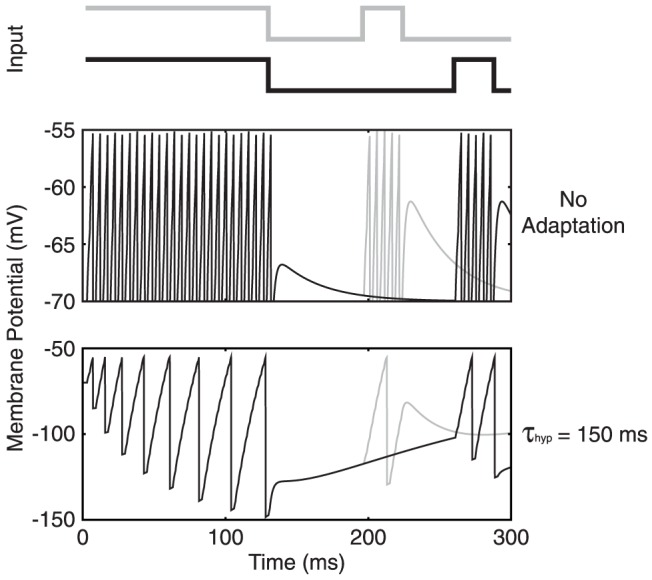
Single neuron gap encoding. Two different input stimuli are shown at the top, with the gray pattern delivering a 64(clipped at 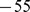 mV) in response to each stimulus is displayed in the middle panel, for a non-adapting neuron. The difference in membrane potential between 64 ms (gray) and 128 ms (black) after the first snippet is not significant enough to result in different spike counts during the second snippet. On the other hand, for an adapting neuron with 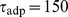 ms, the hyperpolarization and recovery result in a large difference in membrane potential at the two time points such that the neuron produces a different spike count during the second snippet.

We began our simulation study with an example that illustrates how adaptation supports gap discrimination tasks at the single neuron level (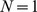). We constructed two noise-free stimuli on a single input fibre with one stimulus containing a 64 ms gap while the other contained an 128 ms gap size. This time, for simplicity, the input snippets for both patterns consisted of periodic input spikes of 500 Hz, where the second snippet was limited to only 30 ms in length for illustration (Figure 5). The choice of 500 Hz input rate here reflects the average input spike rate received per neuron in our network study to be presented later, where 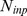  = 1000, 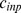  = 0.05 and the spike rate per input fiber is 10 Hz. We first tested the two stimuli separately on a non-adapting neuron and observed its membrane potential over time. While the membrane potential at the onset of the second snippet changed slightly between the two cases, the difference was insignificant such that the two stimuli elicit identical spike counts during the second snippets, failing to encode the different gap sizes in terms of the neuron's spike count.

Conversely, performing the same test on an adapting neuron with 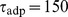 ms resulted in different spike counts between the two stimuli (Figure 5, bottom). In the 64 ms case, the membrane was still much depressed upon the presentation of the second snippet such that only one spike was induced, whereas 128 ms after the first snippet, the membrane had recovered sufficiently such that the second snippet produced two spikes. Gap discrimination was hence achieved by distinct recovery from adaptation.

The greater sub-threshold depression from the shorter gap also means a longer integration time before the neuron reacts with an action potential. This adaptation-based single-neuron model can already help explain the network firing rate behavior observed in Figure 2F. In Figure 2F, the short gaps leave the network substantially hyperpolarized such that its initial firing rate response is in fact below the steady state value. On the other hand, the longer gaps evoke more rapid responses from the cells due to their further recovery.

**Figure 6:**
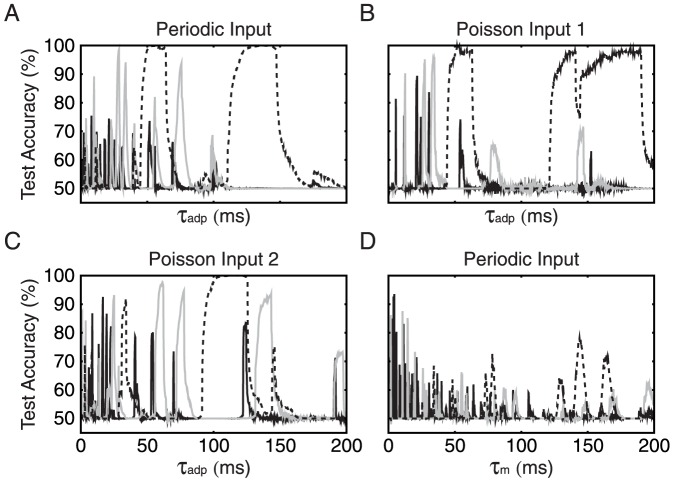
Two-gap classification with a single neuron. (A) Binary classification performance of an adapting neuron for varying adaptation time constant and gap pairs of 4–8 ms (solid black), 16–32 ms (solid gray) and 64–128 ms (broken black). The input patterns are made of 130-and-30 ms snippets, as in Figure 3, containing identical 500 Hz periodic signal spikes. Each input pattern is repeated 10 times against 5 Hz background noise along a single input fiber. (B)–(C) The same experiment as in the top panel, this time with a particular instantiation of 500 Hz Poisson spike train for the input snippets, showing how changing spike timing can alter the peaks. (D) Same experiment as in the top panel, this time varying the membrane time constant 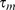 of a non-adapting neuron, as further evidence of the advantage of adaptation in gap detection tasks.

To explore the relevant parameter space of the single neuron example and to quantify how much adaptation aids in gap discrimination, we next applied our binary classification paradigm to the single neuron case. We first restricted the input spike trains to periodic, 500 Hz snippets, and we presented each stimulus 10 times along a single fiber against 5 Hz background noise. The classification results for three different gap pairs are shown in Figure 6A as a function of the adaptation time constant 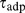, which was used as a free neuronal parameter. Not too surprisingly, the classification performance strongly depended on the gap sizes as well as on the adaptation time constant 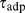. For each gap pair we observed islands of 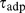 in which the accuracy was well above chance. These accuracy peaks represented regions where the neuron produced different spike counts for the two gap sizes, whereas in the regions outside these peaks the spike counts were the same.

One first notes that smaller gap pairs manifested lower and narrower 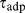 peaks. Hence the task of correctly classifying the 4–8 ms gap pair was not only highly selective in 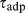 values, but the performance was also very susceptible to noise (Figure 6A). On the other hand, the accuracy curve for the 64–128 ms gap pair exhibited robust performance for a wide range of 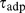 values (Figure 6A). In fact, the broadest peak existed beyond the 200 ms scope in Figure 6A, where the neuron fired once to encode 128 ms gaps and stayed silent for 64 ms gaps.

Furthermore, while the test performance peaks tended to be situated around the order of the gap sizes involved, their widths and distribution were partly sensitive to the makeup of the input snippets. As a validation, we performed the same experiment, this time using two different instantiations of 500 Hz Poissonian snippets to construct our input patterns (Figures 6B–C). We found that, although the islands of high accuracy were at different values of 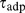, the distribution of these islands were very similar as with periodic input spikes.

Lastly, for comparison, we applied our binary classification paradigm to a non-adapting neuron with variable membrane time constant 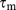. Using the same 500 Hz periodic input snippets, Figure 6D shows that, without adaptation, the neuron performs poorly. The results from Figure 6 therefore suggest that adaptation is a quintessential, and perhaps necessary element in gap discrimination tasks.

From these single neuron simulations, we draw two major conclusions: (1) In order to encode arbitrary gap lengths, we require several neurons with different adaptation time constants, whose islands of high discrimination accuracy cover the whole range of possible gaps lengths. (2) If gap lengths are to be encoded by spike count, the number and size of parameter islands in which spike counts of a single neuron differ for different gap sizes generally improves with an increased number of output spikes. This spike rate may be increased by multiple factors, such as input rate, input connectivity, and recurrent connectivity.

### Gap Discrimination in a Network

As a next step we studied a network of adapting neurons (see Materials and Methods) and investigated how its 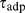 heterogeneity and connectivity parameters influence the classification paradigm.

**Figure 7:**
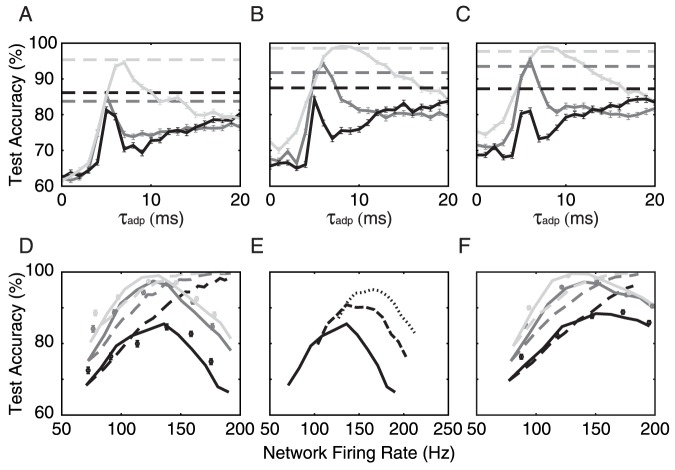
Heterogeneity and recurrence. (A) Network performance is compared between a heterogeneous, non-connected network (broken lines, 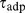 uniformly distributed from 0 to 20 ms) and a non-connected network that is homogeneous in 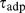 (solid lines), in tasks of classifying 4–8 ms (black), 6–12 ms (dark gray) and 8–16 ms (light gray) gap pairs. 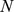  =  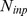  = 1000, 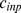  =  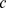  = 0.05, with 10 Hz signal rate and 0.1 Hz noise rate. (B) The same experiment as (A), this time with excitatory recurrence (1.0 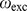, 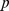  = 0.8), showing classification improvement from a non-connected network. (C) Same experiment as in (B), this time with a mixture of excitatory and inhibitory recurrence (2.0 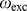 and 0.5 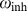), essentially reproducing the improvement seen in (B). (D) Test accuracy as a function of firing rate for heterogeneous adaptation. Network firing rate is tuned by either changing input firing rate (broken lines), starting from 10 Hz signal and 1 Hz noise and keeping signal-to-noise ratio the same, or by changing network recurrent weights, either through pure excitation (solid line) or through exc./inh. mixture (symbols), with 10 Hz signal and 1 Hz noise. The tasks are to classify 8–16 ms (black), 12–24 ms (dark gray) and 16–32 ms (light gray) gap pairs. The effect of network recurrence exhibits a maximum and can be roughly traced either through pure excitation or through exc./inh recurrence, along the network firing rate axis. (E) The 8–16 ms classification task for increasing excitatory recurrence, with input rates of 10 Hz (solid), 20 Hz (long dashed) and 30 Hz (point dashed) and a fixed ratio of background noise of 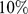 of the input rate. (F) Same as D for networks of neurons with distributed basic properties: Gaussian distribution of membrane time constants 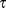 with mean of 30 ms and standard deviation of 15 ms; Gaussian distribution of capacitance 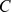 with mean 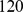 pF and standard deviation 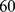 pF.

We first compared heterogeneity to homogeneity in classifying gap pairs of 4–8 ms, 6–12 ms and 8–16 ms with non-connected networks. We constructed the heterogeneous network by randomly choosing each neuron's 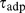 value from a uniform distribution between 0 and 20 ms, and we compare this network's performance to that of a set of homogeneous networks with different constant values of 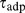. The results are shown in Figure 7A. As expected, the optimum value of 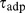 for performing classification in a homogeneous network changed as the gap sizes changed. Also, as far as the linear classifier is concerned, the heterogeneous network provided just as much gap encoding as an optimum homogeneous network. That was because the classifier only required a few units out of the entire population to encode the gaps with high fidelity to correctly perform classification, and in the case of 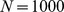 network neurons, a uniform distribution between 0 and 20 ms already supplied a sufficient number of good neurons to equal the performance of a good homogeneous network. Thus, in an environment where the afferent fibers carry a wide variety of gap sizes and spike statistics, it is a viable strategy to achieve good gap discrimination by providing a wide distribution of adapting neurons, such that gap encoding can always be found somewhere within the population response.

**Figure 8:**
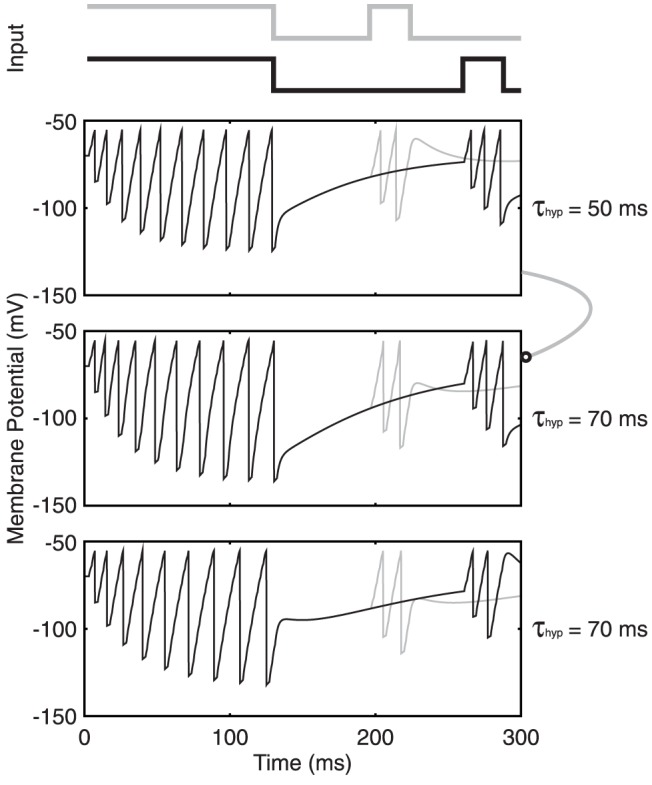
Proliferation of separation. The same paradigm from Figure 5 is employed, with the input snippets connected to three different neurons with 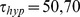 and 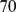 ms. In addition, the top neuron (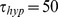 ms) has an excitatory synapse (weight 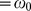) on the middle neuron, resulting in its discriminating firing behavior. The bottom panel shows a stand-alone 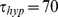 ms neuron that exhibits no discrimination to the two gap stimuli.

We next looked into the advantage of network recurrence by conducting the same experiment, this time with excitatory network recurrence (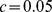, 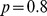, 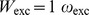 and 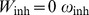). The results, in Figure 7B, indicated that excitatory recurrence enhances a network's ability to create separable patterns to differentiate gaps. One may qualitatively interpret this observation as follows. When input patterns of different gap sizes are presented to a non-connected network, the good neurons will exhibit differential firing while the non-discriminant neurons will produce identical firing counts. Once the neurons are connected, the non-discriminant neurons will receive non-identical numbers of action potentials from the good neurons in response to different gap sizes, creating further separation in the spiking responses of these non-optimum neurons. How a good neuron can proliferate separability to a non-discriminant neuron is illustrated in Figure 8. In our network simulation such proliferation of separation may propagate for a short time before gap information is destroyed by later input spikes, noise and intrinsic network activity.

The aforementioned classification enhancement is not limited to purely excitatory recurrence: The same improvement can also be seen in Figure 7C, where mixed (excitatory/inhibitory) networks were used (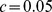, 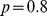, 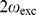 and 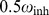). The results from Figures 7B–C hence again suggest that the key parameter governing a population's classification capacity is its firing rate. To prove this, we conducted the same binary classification experiment as above, but this time the input fibers delivered 8–16 ms, 12–24 ms and 16–32 ms gap pairs, while the neurons had a uniform distribution of adaptation time constants 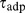 from 0 to 120 ms. We then looked at the network's classification performance as a function of its onset (first 30 ms) firing rate (Figure 7D). Firing rate was changed by either increasing the input rate or recurrent synaptic weights (see caption for details).

The results illustrate that first, network performance scales monotonically with firing rate from the input fibers. This was not surprising, since we expected from the single neuron study (Figure 5) that a higher spike rate along the input fibers triggers higher network spike counts, hence providing more locations along the spike count dimension where separations can be found.

Second, as seen in Figure 7D, the effect of proliferation of separability from a network's recurrence reached a maximum value at roughly the same firing rate for all three gap pairs tested. We interpret this maximum as a point where the strong intrinsic activity starts to generate stereotyped firing patterns that are no longer related to the input features and, as a result, the activity traces induced by the gaps start to become weaker.

The effect of recurrence and the effect of input rate are relatively independent, as is illustrated in Figure 7E, where we raised input rate to the recurrent network in the 8–16 ms task. The incremental effect of increasing input spike rate simply shifts the starting point of the curve to a higher network spike rate and higher performance point, while increasing recurrent weight exhibits the same general behavior, always bringing the performance to a maximum point before deteriorating.

To check how robust our findings were with respect to heterogeneities in the neuron populations we repeated the analysis of Figure 7D for a network with neurons that had capacitance and time constants distributed according to experimental measurements [18] (Figure 7F). Test accuracy shows that such cellular heterogeneity further improves separability of the network patterns for high firing rates, but not for low firing rates.

### Simulated Independent Components

From our previous sections we concluded that heterogeneity in adaptation is a key property of a network to encode gaps in population patterns. We therefore set out to see whether the experimental recordings analyzed by ICA (Figure 3A) were consistent with such heterogeneity. To this end, we fed input patterns of gap sizes 2, 4, 8, 16, 32, 64 and 128 ms to the network, as in the gerbil experiments [15].

We used a network containing a uniform distribution of 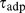 values from 0 ms to 1000 ms, so as to cover all gap sizes, and the recurrent weights for both networks were tuned such that their average onset firing rates match the average onset firing rate of the 91 gerbil neurons measured (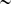 30 Hz). We set the mean Poisson input spike rate to be 10 Hz, against 1 Hz background noise, and use 130 ms snippets to construct our input patterns constructed from ten unique snippet pairs (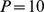). Each input pattern was repeated 10 times. We then followed the same ICA steps as done for the experimental data by collecting the network's response during each second snippet (10 ms bins) and looked for the most prominent independent components.

**Figure 9:**
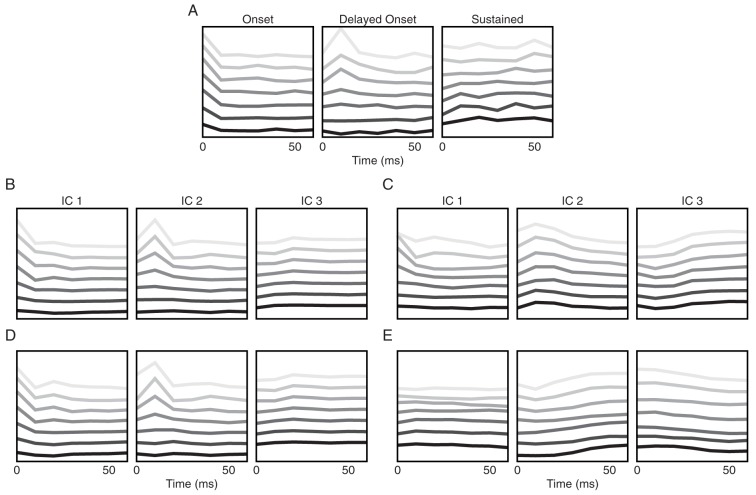
Independent components of network simulations. (A) Independent components from experiment. Replotted from Figure 1E for comparison. (B) Independent components of a heterogeneous recurrent network. The gap sizes (gray levels: dark to bright means 2 ms to 128 ms) are the same as those presented to the gerbils in [15], and the network contains 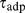 values uniformly distributed from 0 to 1000 ms. Recurrent weights (4 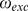 and 4 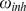) are tuned such that the network's onset firing rate matches that of the gerbil inferior colliculus neurons (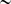 30 Hz). (C) Independent components from a recurrent, homogeneous network of 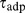  = 50 ms (4 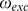 and 12 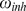). (D) Independent components from a non-connected, heterogeneous network (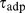 between 0 and 1000 ms). An input rate of 9 Hz with 0.9 Hz noise rate were needed to achieve 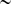 30 Hz of network firing rate. (E) Independent components from a non-adapting recurrent network (4 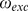 and 28 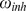).

The results for the heterogeneous recurrent network are shown in Figure 9B. For comparison, we performed the same study on a recurrent homogeneous network (Figure 9C), a non-connected heterogeneous network (Figure 9D) and a recurrent non-adapting network (Figure 9E). All networks were tuned to the same firing rate (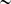 30 Hz). We found that the two heterogeneous networks manifested qualitatively the same onset, delayed onset, and sustained components as observed from the gerbil inferior colliculus neurons. Functionally, in this multi-gap classification task, the heterogeneous recurrent network (Figure 9B) performed slightly better than the non-connected heterogeneous network (Figure 9D; 67.4% vs. 64.2% test accuracies), followed by the homogeneous adapting network (Figure 9C; 61.8%). Lastly, the non-adapting network (Figure 9E) displayed distinctly inferior accuracy (38.6%) than its adapting counterparts.

Figure 9D shows that the three dominant ICs can be observed without the effect of intrinsic connectivity. This implies that the onset, delayed onset, and sustained patterns arise from individual neurons. Recalling our single neuron study, we imagine that the various input patterns are processed by all neurons along the 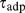 axis, eliciting onset response from some, delayed onset response from some others, and sustained responses from yet some other neurons. When one homogenizes the adapting network, the diversity along the 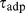 axis is lost, and hence so is the variety of response types. This is shown in Figure 9C, where a homogeneous network of 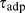  = 50 ms only renders delayed onset and sustained patterns. Lastly, the non-adapting network essentially contains only sustained components, which provide scant gap-encoding capacity, as evidenced by its poor classification performance (cf. Figure 7A for 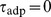).

In summary, our results thus indicate that (1) gap encoding in the gerbil inferior colliculus is consistent with heterogeneity in adaptation, and that (2) this encoding is best achieved at moderate recurrent drive from the network.

### Invariance of the Gap Code

**Figure 10:**
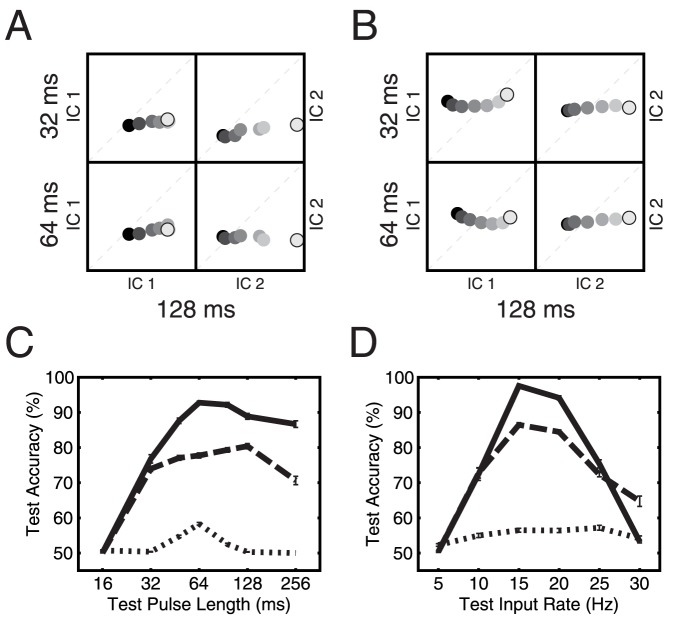
Invariance of the gap code. (A) Projections 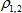 on the first two independent components of the gerbil recordings (128 ms pulse length): projections of activity from reduced pulse lengths (32 and 64 ms as indicated) vs. original (128 ms pulse length). Dark dots indicate short gap lengths, bright dots indicate long gaps. Dashed lines indicate identity. (B) Same as A for simulations of the network from Figure 9B. (C) Test accuracy of a linear classifier for gap discrimination trained on the simulated network from B for multiple pulse lengths (32, 64, and 128 ms). Gap pairs were 128 ms vs. 64 ms (solid line), 64 ms vs. 32 ms (dashed line), and 8 ms vs. 4 ms (dotted lines). (D) Test accuracy of a linear classifier for gap discrimination trained on the simulated network from B for multiple input rates (10, 15, 20 Hz). Gap pairs as in C.

We finally asked, whether the observed population activity patterns not only encode gaps within the tight constraints of our paradigm, but also show some degree of invariance against changes of the parameter regime. First, we analyzed the gerbil data obtained for the same gap sizes but with varying durations of the preceding noise pulses and compared their projections onto the onset and delayed onset ICs (from 128 ms pulses) to projections of the original 128 ms pulses. For short gap sizes (dark dots), the independent components capturing onset and delayed onset responses were relatively invariant with respect to pulse length (Figure 10A). Deviations from invariance occurred for longer gap sizes (brighter dots) and were relatively gradual and systematic such that a downstream station could easily achieve invariant decoding by a linear transformation. This finding was not necessarily unexpected, since gap length discrimination was shown to depend on pulse length in human psychophysics as well [21].

Next, we did the same analysis for our simulated network with heterogeneous adaptation and excitatory and inhibitory recurrent couplings. Also there, onset and delayed onset components showed gradual and systematic deviations (Figure 10B). Particularly the behavior of the delayed onset component (IC2) matches that of the physiological data well. To test invariance from a functional perspective, we then used the linear classifier and trained it with pulse lengths of 32, 64 and 128 ms, before testing it with a whole range of pulse lengths between 16 and 256 ms (Figure 10C). The test accuracy was almost invariant for pulse lengths of 32 ms and larger, verifying that invariance can be functionally extracted from IC patterns, at least for some of the pulse lengths. As a last test we also varied the firing rate by first training the classifier with input rates of 10, 15 and 20 Hz and found that the classifier works well (Figure 10D) in a relatively broad range of input rates (10 to 25 Hz).

From these tests, we conclude that heterogeneous adaptation allows a linear classifier to extract gap durations with some degree of invariance to pulse lengths and background rate and thus likely provides a robust code for gap size that only changes gradually with variations of the stimulus paradigm.

## Discussion

We investigated gap encoding in the inferior colliculus, through both analysis of experimental data from gerbils and simulation of neural networks. Our independent component analysis revealed that, when presented with stimuli containing multiple gap sizes, neurons responded with three prominent population patterns: onset, delayed onset, and sustained. Only the onset and delayed onset components showed gap-encoding capacity. In our computational effort to understand gap processing in inferior colliculus, we employed a simple input/network/read-out paradigm that emulated some of the basic features of the auditory midbrain. Then, starting from a single adapting neuron, we showed that experimentally-observed population patterns could arise from heterogeneous adaptation in a network. Moreover, network recurrence could serve to further enhance the network's ability to provide discriminable population patterns.

Psychophysical experiments in gerbils [22] and rats [23] show gap detection thresholds as short as a few milliseconds. This finding imposes a strong constraint on the shortest adaptation time-scales in the model. Gap *discrimination* tasks in rodents are rather rare. In [23], it was shown that rats can learn to distinguish a 15 ms from a 60 ms gap, which could be easily explained by the differences in the independent components from our gerbil recordings. In [24] gap discrimination in rats was measured for two reference gap sizes (15 and 40 ms). For both gap sizes the relative gap discrimination error was about 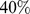. These results are also in rough qualitative agreement with the clearly observable differences in the (gerbil) independent components for gap sizes of 8, 16, 32, and 64 ms (Fig. 1E).

The inferior colliculus is a very heterogeneous brain structure, morphologically and physiologically [25], in terms of its inputs [7], but, most prominently, in terms of its responses. Some neurons' responses are very specifically related to the ethology of the animal such as breath-selective [26] or wingbeat-specific [27] neurons. Some are more general responses that are simple combinations of elementary receptive fields such as duration tuned neurons [28], target-distance-specific responses in echolocating bats [29], or combinations of temporally segregated frequencies [30], [31].

In light of this variety of receptive fields, it may not come as a surprise that there are only few general theories on inferior colliculus function. One of these theories [4], suggests two general types of inferior colliculus responses. One type of receptive fields contains stimuli that are essential for the survival of animals and the outputs are directly conveyed to the motor system (for example neurons that are selective to wing-beat patterns of prey). These receptive fields have to be very specific and detailed. The other type of receptive fields are rather general and unspecific (e.g. combination-specific neurons) and can be seen as multi-purpose primitives that are useful to further cortical processing. For both response types the downstream stations (motor and cortical) operate on a slower time scale than that of the auditory input and thus the inferior colliculus has to encode information in rate (or population pattern).

The translation from time to rate can occur by means of adaptation (as in our model) but can also result from intricate combinations of inhibition and excitation via delay lines [32], a degree of freedom we have neglected in this paper. While we have focused most of our attention on the heterogeneity in adaptation time constants, there are several other conceivable mechanisms that would generate an analogous effect. One such mechanism is the initial amplitude of the adapting hyperpolarization, 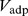, since, at the single neuron level, the slope of the recovery in membrane potential (Figure 3) linearly scales with 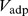. Thus, in principle the heterogeneity in adaptation slopes could also be achieved by a heterogeneity in adaptation strengths 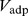. Also different levels of delayed feed-forward inhibition can generate a heterogeneity in re-depolarization time courses, which would have the same effect as the heterogeneity in adaptation time constants. For the present study, we chose to only explore 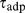 in an effort to coarse-grain our investigation of heterogeneous adaptation. In the bigger picture, we expect each neuron's membrane behavior to be a function of all neuronal parameters as well as the external inputs: heterogeneity may arise along all pertinent parameter dimensions to optimize the network's performance. This idea, of course, also pertains to other nuclei that have been suggested to contribute to gap encoding, such as the paraolivary nucleus [33], for example, via heterogeneity of its postinhibitory rebound spikes.

Our model can be generalized to also describe population coding of amplitude-modulated (AM) signals. Neurons in the inferior colliculus discharge phase-locked to AM stimuli [34], [35]. Intrinsic neuronal properties inducing adaptation effects have been shown to strongly influence single unit phase-locking in a model [36]. Our results predict that, beyond single unit responses, population patterns are also highly informative about AM frequency due to the heterogeneous cellular adaptation time constants.

Adaptation is ubiquitous along sensory pathways [37]–[41] and there are several specific accounts of its functional role related to the processing of temporal stimulus features [42]–[44]. Also the benefits of heterogeneity have already been studied [45]. This paper proposes a further mechanism, both at the single neuron level and at the network level, of how adaptation provides improved discriminability of temporal gaps and selective processing of amplitude modulations in an auditory stimulus. Beyond the auditory system, our model can be generalized to other modalities. For example, in the visual domain, spatial motion can be interpreted as the movement of brightness patches, which translates to amplitude modulations of brightness at one retinal location.
